# Music and Visual Art Training Modulate Brain Activity in Older Adults

**DOI:** 10.3389/fnins.2019.00182

**Published:** 2019-03-08

**Authors:** Claude Alain, Aline Moussard, Julia Singer, Yunjo Lee, Gavin M. Bidelman, Sylvain Moreno

**Affiliations:** ^1^Rotman Research Institute, Baycrest Centre for Geriatric Care, Toronto, ON, Canada; ^2^Centre de Recherche de l’Institut Universitaire de Gériatrie de Montréal, Université de Montréal, Montréal, QC, Canada; ^3^Institute for Intelligent Systems – School of Communication Sciences and Disorders, The University of Memphis, Memphis, TN, United States; ^4^Digital Health Hub, School of Engineering Science, Simon Fraser University, Surrey, BC, Canada

**Keywords:** aging, music training, art training, brain plasticity, executive functions, ERPs

## Abstract

Cognitive decline is an unavoidable aspect of aging that impacts important behavioral and cognitive skills. Training programs can improve cognition, yet precise characterization of the psychological and neural underpinnings supporting different training programs is lacking. Here, we assessed the effect and maintenance (3-month follow-up) of 3-month music and visual art training programs on neuroelectric brain activity in older adults using a partially randomized intervention design. During the pre-, post-, and follow-up test sessions, participants completed a brief neuropsychological assessment. High-density EEG was measured while participants were presented with auditory oddball paradigms (piano tones, vowels) and during a visual GoNoGo task. Neither training program significantly impacted psychometric measures, compared to a non-active control group. However, participants enrolled in the music and visual art training programs showed enhancement of auditory evoked responses to piano tones that persisted for up to 3 months after training ended, suggesting robust and long-lasting neuroplastic effects. Both music and visual art training also modulated visual processing during the GoNoGo task, although these training effects were relatively short-lived and disappeared by the 3-month follow-up. Notably, participants enrolled in the visual art training showed greater changes in visual evoked response (i.e., N1 wave) amplitude distribution than those from the music or control group. Conversely, those enrolled in music showed greater response associated with inhibitory control over the right frontal scalp areas than those in the visual art group. Our findings reveal a causal relationship between art training (music and visual art) and neuroplastic changes in sensory systems, with some of the neuroplastic changes being specific to the training regimen.

## Introduction

Aging is associated with structural and functional changes in prefrontal, parietal, and medial temporal regions, which has been linked to enhanced sensory evoked responses in auditory and visual areas as well as deficits in attention, memory and executive functions (Dempster, 1992; Alain and Woods, 1999; West and Alain, 2000; Bugaiska et al., 2007; Kropotov et al., 2016; Aljondi et al., 2018). Some theories emphasize age-related declines in inhibitory processes (Hasher and Zacks, 1988; Knight, 1994; West, 1996), which involve the ability to regulate incoming sensory input and behavior so as to override internal predispositions or external lures to accomplish more appropriate actions or behaviors (Diamond, 2013). Age-related declines in attentional regulation and inhibitory control (May et al., 1999; Erber, 2013) result in negative consequences for perceptual and cognitive skills (Gazzaley et al., 2007; Lustig et al., 2007), which may be reflected in the overall responsiveness (amplitude) of sensory evoked responses (Bidelman et al., 2017). Age-related declines in inhibitory control may also be associated with impairments in language comprehension (Pichora-Fuller et al., 1995, 2017) and can lead to an increase in socially inappropriate behavior. Given the rapid expansion of the aging population in modern society, there is increasing need to identify and develop effective interventions that reduce, halt, or even reverse declines in inhibitory control to preserve daily cognitive abilities in older adults.

Recent studies have shown that lifelong engagement in musical activities might help maintain the brain in a younger state (Rogenmoser et al., 2018), slow cognitive declines (Hanna-Pladdy and Gajewski, 2012; Parbery-Clark et al., 2012; Zendel and Alain, 2012; Bidelman and Alain, 2015), and preserve or even enhance neural functioning in old age (Parbery-Clark et al., 2011; Bidelman et al., 2014; Zendel and Alain, 2014). For example, older musicians show improved performance compared to non-musicians in several cognitive areas including spatial memory, processing speed, and cognitive flexibility (Parbery-Clark et al., 2011), as well as inhibitory control (Moreno et al., 2014; Moussard et al., 2016). A positive relationship between years of musical engagement and cognitive performance further implied experience-dependent changes in older adults’ behavioral skills (Parbery-Clark et al., 2011, 2012; Bidelman and Alain, 2015).

Several developmental studies have been conducted investigating the effect of learning how to sing or to play a musical instrument over a period of time. Using expert and intervention designs, results have shown benefits on different types of cognitive processes such as verbal processing (Moreno et al., 2009; Seither-Preisler et al., 2014), intelligence (Schellenberg, 2004), reading (Moreno et al., 2011), inhibitory processing (Moreno et al., 2011), auditory processing (Moreno and Farzan, 2015; Tierney and Kraus, 2015) and on general brain development (Hyde et al., 2009). However, evidence to date demonstrating benefits of musical training in older adults has often been correlational, not causal. Very few studies have investigated, in longitudinal designs, whether engagement in musical activity can improve cognitive functions. Although evidence suggests that short-term musical training (e.g., piano lessons) can improve executive functioning and working memory (Bugos et al., 2007; Seinfeld et al., 2013), prior studies often suffer from methodological limitations (e.g., lack of an active control group, lack of randomization for group assignment), and have not measured the effects of the intervention on brain activity. Thus, it remains unclear whether engagement in musical activities would yield neuroplastic changes in brain activity, and whether these changes could be long-lasting. Examining neural activity prior to, and after short-term music training and comparing it with that of another artistic activity would allow for a greater understanding of brain plasticity and potential transfer mechanisms in the aged brain.

A cognitively demanding form of training that could be considered equally as engaging as musical activity is visual art. Evidence from neuroimaging studies suggests that activity elicited during visual art engagement, including both visual object learning (Op de Beeck and Baker, 2010) and hand-related motor activities (Draganski et al., 2004), induces neuroplastic changes in brain areas associated with spatial-reasoning skills (Pollmann and von Cramon, 2000). Visual art training has also been associated with structural differences in areas of the brain pertaining to fine motor control and procedural memory between artists and non-artists (Chamberlain et al., 2014). Therefore, there is reason to believe that visual art training, like musical training, may promote cognitive improvements in older adults. Importantly, visual art training provides a means to assess whether neuroplastic changes associated with music training are specific to music training itself or whether they reflect non-training specific effects associated with engagement in art programs in general.

In this study, we investigated the short-term (3-month) impact of two forms of engaging training – music and visual art instruction – on older adults’ perceptual and cognitive functions using a three group intervention design (music, visual art and a no-contact control). We also measured maintenance of the effects with a follow-up testing 3 months after the cessation of training. All participants completed a brief neuropsychological assessment to ensure that our groups were comparable before training. We used the same battery after training and at follow-up to explore whether music and visual art training impacted cognitive functioning as measured with neuropsychological tests. We anticipated that participants in the music group would show higher cognitive functioning than those in the active (visual art) and passive control groups. We anticipated that participants in the music group would show higher cognitive functioning after training than those in the control group. We also expected potential improvements following visual art training, as an exploratory hypothesis.

In addition, we recorded neuroelectric event-related potentials (ERPs) using auditory oddball paradigms and during a visual GoNoGo task at each session (pre, post, and follow-up), to evaluate training-related neuroplastic changes in sensory processing and executive functions. These paradigms were chosen because prior studies have shown neuroplastic changes associated with music training on these tasks (e.g., Shahin et al., 2003; Zendel and Alain, 2009; Moussard et al., 2016). We hypothesized that each form of art training would impact sensory processing in the trained modality paralleling training-related benefits reported in children (e.g., Fujioka et al., 2006; Moreno et al., 2011, 2014). That is, music training was expected to impact the N1 and P2 waves of the auditory ERPs as previously found in younger populations (e.g., Tremblay et al., 2001; Reinke et al., 2003; Alain et al., 2010). We also anticipated that music training would improve listeners’ ability to notice changes in auditory stimuli as indexed by the mismatch negativity (MMN), which has also been observed in young adults (Tervaniemi et al., 1997; Nikjeh et al., 2009). We also hypothesized that visual art training would have greater impact than music training on visual sensory evoked responses such as the N1 and P2 waves at parietal-occipital sites.

Furthermore, we expected to observe neuroplastic enhancements in brain mechanisms subserving inhibitory control in the music group relative to the visual art group and age-matched no-training controls. Following music training, changes were expected in the N2, and P3 complex of the ERPs during the visual GoNoGo task as previously found in younger (Brydges et al., 2014; Moreno et al., 2014) and older (Moussard et al., 2016) populations. As shown in previous studies in young adults, music training induced enhancements in right hemisphere activity (Moreno et al., 2014). Thus, we hypothesized that the music group would show an increase in ERP amplitude over the right hemisphere after training. Prior research has also shown long lasting benefits of music training that persist years after the training has ended (White-Schwoch et al., 2013). Hence, we expected brain and behavioral enhancements to persist at 3-month follow-up. This finding would establish that relatively engaging art training could produce lasting effects in neural and behavioral function in older adults well after the cessation of instruction.

## Materials and Methods

### Participants

Sixty healthy older adults with limited prior musical or visual art training were recruited from the Greater Toronto Area. For pre- and post-training phases, five participants were lost to attrition, and two due to technical problems during EEG recording, resulting in 17 participants in the music group (three males), 19 in the visual art group (two males), and 17 in the no-contact control group (three males). The groups did not differ in age (*p* = 0.82; music: *M* = 67.7, *SD* = 5.8 years; visual art: *M* = 68.9, *SD* = 6 years; control, *M* = 68.5, *SD* = 6 years), years of formal education (*p* = 0.55; music: *M* = 16.4, *SD* = 2.6 years; visual art: *M* = 17.2, *SD* = 2.3 years; control: *M* = 16.9, *SD* = 1.5 years), or intelligence on the Wechsler Abbreviated Scale of Intelligence-Second Edition (WASI-II FSIQ4; *p* = 0.51; music: *M* = 114.2, *SD* = 10.5; visual art: *M* = 115.8, *SD* = 11; control: *M* = 111.8, *SD* = 13.9). Participants were screened for amusia and other auditory or musical deficits that could have interfered with the study using the Musical Ear Test (MET, Wallentin et al., 2010). All three groups showed similar scores at baseline psychometric assessment (all *p*-values > 0.1). After 3 months, 15 participants from the music group and 14 participants from the visual art group returned for follow-up testing. These two subgroups remained similar in age (*p* = 0.57), education (*p* = 0.31), and had comparable intelligence on WASI-II FSIQ4 (*p* = 0.39) at pre-test. The study received approval from the Baycrest Research Ethics Committee, and all participants provided written informed consent.

### Study Design

**FIGURE 1:**
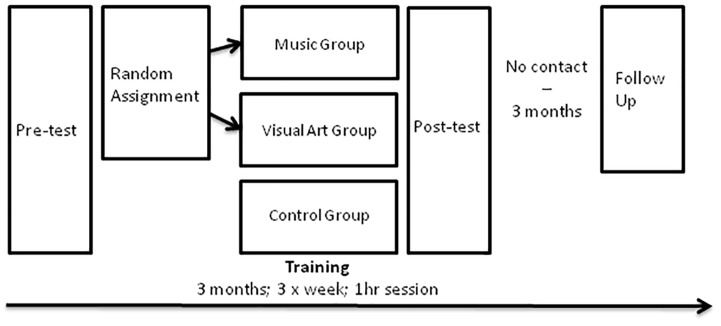
Schematic of the study design.

This longitudinal study consisted of four phases: pre-test, 3-month training, post-test, and 3-month follow-up test (Figure 1). During the 3 months between post-test and follow-up, participants did not engage in formal music or visual arts activities. At pre-test, post-test, and follow-up sessions, participants were tested individually and were blind to our hypotheses. After the pre-test, participants were assigned to either music or visual art training in a pseudorandom manner to equate pre-training differences between groups on intelligence scores and background demographic measures (gender, age, and years of education). An additional passive control group was further recruited in order to collect data to distinguish potential training effects from test–retest effects.

### Training Curricula

The trained participants received group classroom instruction and activities in their respective training by a professional teacher (i.e., two teachers: a music and a visual art teacher) at the Royal Conservatory of Music in Toronto for 3 months (36 1-h sessions, three times per week). The music group was engaged in music making using body percussion, voice, and non-pitched musical instruments. They also learned basic music theory as well as melody and harmony concepts through the singing of simple canons. The visual art group learned basic drawing and painting techniques, analyzed work of famous artists, and created original paintings of landscape, still-life, and self-portrait. All materials were provided for them (i.e., instruments for music lesson and all the material for drawing and painting in the visual art lesson).

### Procedure

Testing (EEG and psychometric tests) took place in the laboratory on two different days and lasted approximately 1.5–2 h per session. For EEG testing, recordings took place in an acoustically and electrically shielded room.

#### Psychometric Assessment

##### Wechsler Abbreviated Scale of Intelligence-Second Edition (WASI-II)

WASI-II assesses intelligence using four different subtests including measures of verbal comprehension: (1) Vocabulary and (2) Similarities; and measures of perceptual reasoning: (3) Block Design and (4) Matrix Reasoning. A composite score (FSIQ4) was then calculated to reflect global IQ.

##### Forward and Backward Word Span

The Word Span task measured verbal working memory, with one *forward* and one *backward* condition. The experimenter read sequences of words with a 1-s pause between words. Word lists were organized into sequence lengths (*spans*) of two to eight. There were two versions of the test that were counterbalanced across testing sessions. Each sequence had two trials. Testing continued until the experimenter recorded all recalled words from each list. A total span score was calculated for each condition as the total number of correctly recalled items across all trials (maximum *word span* score = 70).

##### Stroop Test

A paper version with three subtests of the Stroop test was used to measure cognitive inhibition and processing speed (Stroop, 1935). Subtest 1 involved reading color words printed in black font while subtest 2 included naming the color of squares. Subtest 3 required naming the color in which the word was printed while the written color name differed (i.e., incongruent condition). Response time was measured for each subtest. The interference score was calculated by subtracting response time on subtest 3 from that of subtest 2. The interference effect was also reflected by the number of mistakes at subtest 3.

##### Computerized Peabody Picture Vocabulary Test (PPVT)

The Peabody Picture Vocabulary Test (PPVT) was used to measure receptive vocabulary (Dunn and Dunn, 2007). In this test, participants saw four pictures on the screen while the computer said a word. Their task was to click on the picture that best characterized the meaning of the word. We used the number of correct responses in the analysis.

##### Digit symbol (subtest of the WAIS-R)

Participants were given 2 min to copy symbols below numbers according to a coding key where a number corresponded to a specific symbol. The test was scored as the number of correctly completed symbols.

#### EEG Recording and Data Analysis

To probe training-related changes in brain activity supporting perceptual and executive control processes, we measured neuroelectric activity (electroencephalogram, EEG) using auditory oddball paradigms and during a visual GoNoGo task. The same stimuli were used at each of the three testing sessions (pre, post, follow-up) while the order of trials was randomized across participants and sessions.

##### Auditory oddball paradigms

Auditory processing was assessed via the N1, P2 and mismatch negativity (MMN) recorded before and after training using two oddball sequences consisting of a either music or speech sound contrast. For the music condition, two synthesized piano tones, Eb4 (F0 = 314 Hz) and D4 (F0 = 294 Hz) served as the standard and deviant tokens, respectively. The two notes differed only in pitch and were otherwise matched in acoustic characteristics including overall amplitude and duration (500 ms). For the speech condition, two French vowels as produced by a native female speaker, /u/ and /ou/, functioned as the standard and deviant stimulus, respectively. The two speech tokens had similar duration (280 ms), mean voice fundamental frequency (F0: ∼240 Hz), amplitude, and first/third formant frequencies; only their second formant differed to yield the two distinct vowel timbres (/u/: ∼1,850 Hz; /ou/: ∼750 Hz). Presentation order was pseudorandom such that at least one standard stimulus preceded a deviant. Stimuli were presented with a stimulus onset asynchrony of 750 ms and delivered binaurally via ER-3 insert earphones (Etymotic Research) at an intensity of 80 dB sound pressure level. A total of 510 standards and 90 deviants (i.e., 85/15% ratio) were collected for both the speech and music conditions.

##### Visual GoNoGo task

Participants were presented with white or purple geometric triangle or squares at the center of the screen located at about 1 m from the participant. Before each trial, a white fixation cross appeared on a black background for a variable duration (500–1,000 ms), then a geometric shape appeared in the center of the screen for 500 ms. Participants were instructed to press the right mouse button in response to white shapes (80% probability) and to withhold responding to purple shapes (20% probability). The experiment consisted of 200 trials (160 Go and 40 NoGo trials). A practice block of 20 trials was used to familiarize participants with the task. During the task, participants did not receive feedback on their performance. Accuracy rates were recorded for Go and NoGo trials, and reaction times were recorded for Go trials.

##### EEG recording and data processing

EEG was recorded from 66 scalp electrodes using a BioSemi Active Two acquisition system (BioSemi V.O.F., Amsterdam, Netherlands). The electrode montage was according to the BioSemi electrode cap based on the 10/20 system and included a common mode sense active electrode and driven right leg passive electrode serving as ground. Ten additional electrodes were placed below the hair line (both mastoid, both pre-auricular points, outer canthus of each eye, inferior orbit of each eye, two facial electrodes) to monitor eye movements and to cover the whole scalp evenly. The latter is important because we used an average reference (i.e., the average of all scalp EEG channels as the reference for each EEG channel) for ERP analyses. Neuroelectric activity was digitized continuously at rate of 512 Hz with a bandpass of DC-100 Hz, and stored for offline analysis. Offline analyses were performed using Brain Electrical Source Analysis software (BESA, version 6.1; MEGIS GmbH, Gräfelfing, Germany).

Continuous EEGs were first digitally filtered with 0.5 Hz high-pass (forward, 6 dB/octave) and 40 Hz low-pass filters (zero phase, 24 dB/octave). For the auditory evoked potentials, the analysis epoch consisted of 100 ms of pre-stimulus activity and 500 ms of post-stimulus activity time-locked to sound onset. For the GoNoGo, the analysis epoch consisted of 200 ms of pre-stimulus activity and 1,000 ms of post-stimulus activity time-locked to the onset of the visual stimuli. EEG segments contaminated by blink and saccade were corrected using BESA. Both eye blinks and lateral movements were first identified in the continuous EEG and then modeled using artifact correction with a surrogate model. After ocular correction, traces were then scanned for artifacts and epochs including deflections exceeding 120 μV were marked and excluded from the analysis. The remaining epochs were averaged according to electrode position, trial type (e.g., Go, NoGo), and session (i.e., pre-training, post-training, follow-up). Each average was baseline-corrected with respect to the pre-stimulus interval.

For the oddball paradigms, the proportion of trials included in the auditory ERPs ranged from 283 to 510 trials for the standard stimuli and from 48 to 98 for deviant stimuli. The number of trials was comparable for the pre- (Standard: *M* = 487; Deviant: *M* = 85) and post-training sessions (Standard: *M* = 484; Deviant: *M* = 86), and was similar for controls (Standard: *M* = 493; Deviant: *M* = 88), music (Standard: *M* = 478; Deviant: *M* = 84) and visual art (Standard: *M* = 484; Deviant: *M* = 85) groups. For the GoNoGo task, the number of trials included in the visual ERPs ranged from 78 to 160 trials for the Go condition and 17 to 40 trials for the NoGo condition. As for the oddball paradigms, the number of trials was comparable before (Go: *M* = 141; NoGo: *M* = 34) and after (Go: *M* = 144; NoGo: *M* = 35) training, and was similar for control (Go: *M* = 148; NoGo: *M* = 36), music (Go: *M* = 141; NoGo: *M* = 33), and visual art (Go: *M* = 139; NoGo: *M* = 34) groups.

##### Statistical analyses

Training effects on the psychometric measures were assessed using repeated measures ANOVAs. We focused on interactions that involved group and session as factors. Although they were not central to the goal of the present study, we also report other main effects and interactions for sake of completeness. For the psychometric assessments, we used the Benjamini–Hochberg method to adjust the familywise *p*-value for multiple comparisons (*q* = 0.1, *m* = 30, and *p* = 0.05), resulting in a *p*-value of 0.005 for significance.

The ERP analyses focused on pre-defined time windows and electrode clusters motivated by prior studies (e.g., Reinke et al., 2003; Moreno et al., 2014; Moussard et al., 2016). For the oddball paradigms, the effects of training on N1 and P2 deflections was assessed during the 90–130 ms and 170-210 ms interval at fronto-central scalp sites (F1, Fz, F2, FC1, FCz, FC2, C1, Cz, C2). The automatic change detection was quantified using the difference waves between standard and deviant. The peak amplitude and latency was quantified during the 100–250 ms window using the same electrode clusters as the N1 and P2 waves. For the GoNoGo task, the processing of go and nogo stimuli was assessed during the 165–205 ms interval (i.e., N1) and the 210–280 ms interval at parieto-occipital electrodes from the left (PO7, P5, P7) and right hemisphere (PO8, P6, P8). The effects of music and art training on the ability to inhibit a motor response on the nogo trials was quantified by comparing mean amplitude for the 375–475 ms interval measured over the left (F1, F3, AF3) and right (F2, F4, AF4) frontal scalp regions. These electrode clusters were chosen because music training in young adults has been shown to modulate visual GoNoGo ERP amplitude over the right hemisphere (Moreno et al., 2014). Thus, we anticipated that music activities in older adults would also show changes in ERP amplitude over the right hemisphere after training.

The effects of art training on auditory evoked responses (i.e., N1, P2, MMN) were assessed using a mixed model ANOVA using groups (control, music, visual art) as between-subject factor, and sequence type (music vs. speech) and stimulus type (standard, deviant) as the within-subject factors. The effects of art training on visual evoked responses recorded during the GoNoGo task was assessed using a mixed model ANOVA with group (control, music, visual art) as the between-subject factors, and stimulus type (Go, NoGo trials) and hemisphere (right, left) as within-subject factors. We used the Benjamini–Hochberg method to adjust the familywise *p*-value for multiple comparisons with *q* = 0.1, *m* = 160 (i.e., number of *p*-values) and *p* = 0.05 (Hochberg and Benjamini, 1990), resulting in a *p*-value of 0.025 for significance.

## Results

### Psychometric Assessment: Training Effects

**FIGURE 2:**
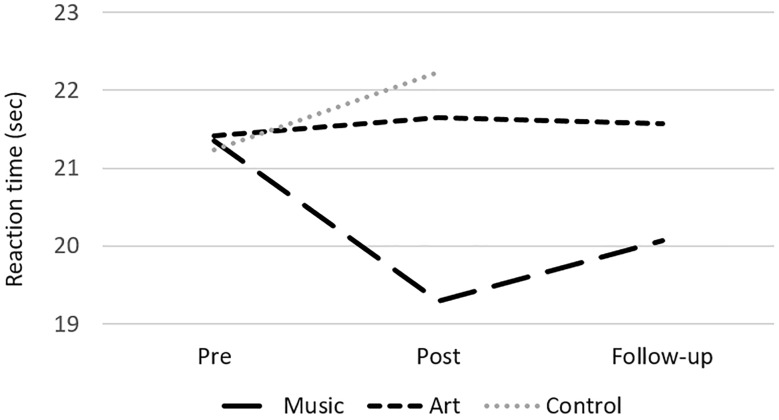
Mean reaction time at the Stroop subtest 2 (color naming) for the music, art, and control groups at pre-test, post-test and follow-up.

The analyses of performance at neuropsychological test revealed a group × session interaction on the Stroop subtest 2 [color naming speed; *F*(2,50) = 6.16, *p* = 0.004, η^2^ = 0.20], where the music group improved in naming speed between pre- and post-assessment [*t*(16) = 3.32, *p* = 0.004, Figure 2]. There was also a main effect of session for the Digit Symbol task, with better performance at post-test [compared to pre-test; *F*(1,50) = 15.98, *p* < 0.000, η^2^ = 0.24], but no interaction with group. There were no other significant differences in performance between pre- and post-training session. In the music group, a follow-up analysis showed that the gain in response speed was maintained 3 months after training (post-test vs. follow-up contrast, *p* > 0.1).

### Effects of Music and Visual Art Training on Auditory Processing

**FIGURE 3:**
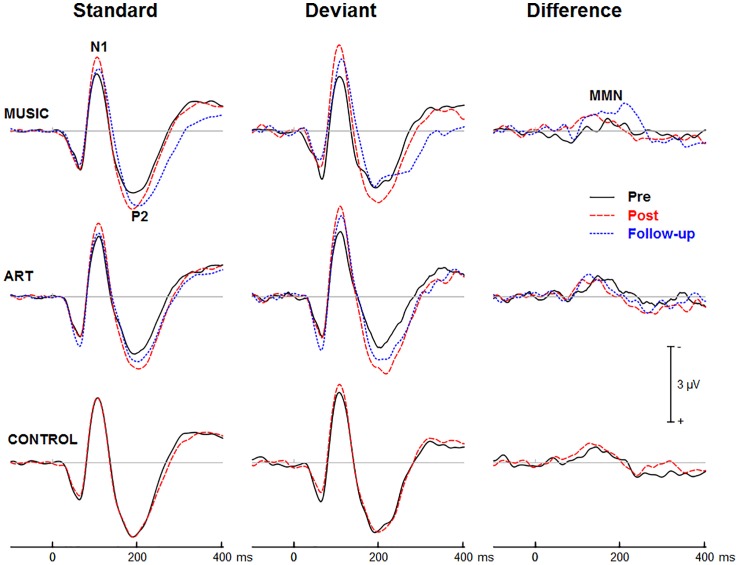
Group mean event-related potentials elicited by standard and deviant piano tones as well as the corresponding difference wave (i.e., MMN). Here and throughout, negativity is plotted upward. Waveforms are from the midline fronto-central electrode (FCz).

Figure 3, 4 show group mean auditory ERPs elicited by standard and deviant stimuli as well as the corresponding difference waves in both sequences (piano tones and vowels) at pre- and post-training sessions, as well as from the subset of participants who completed the follow-up. The scalp-recorded ERPs comprised prototypical deflections at ∼60, ∼110, and ∼195 ms after sound onset (i.e., P1-N1-P2). The MMN was isolated as the difference in neural activity between standard and deviant (oddball) stimuli. One participant from the music group and two from the art group were excluded from the analyses because data from one of the two sequences were unavailable due to technical problems.

**FIGURE 4:**
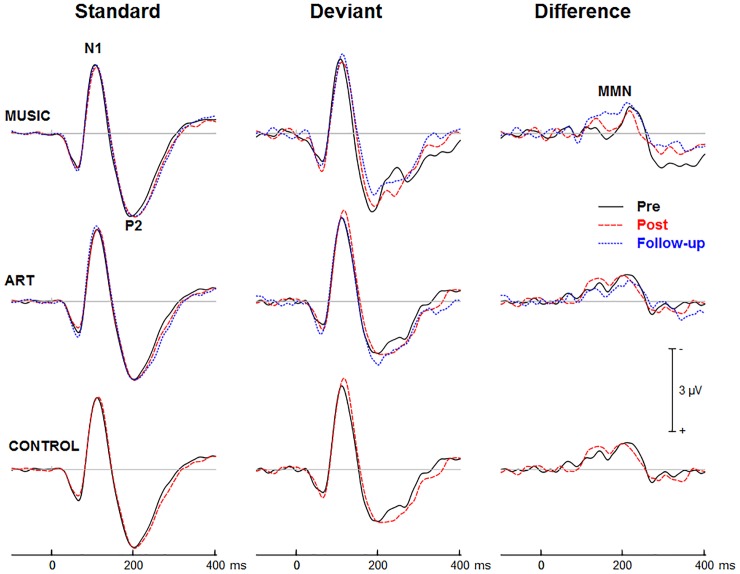
Group mean event-related potentials elicited by standard and deviant vowel stimuli as well as the corresponding difference wave (i.e., MMN). Waveforms are from the midline fronto-central electrode (FCz).

#### Training Effects on Auditory ERPs

First, we examined whether music and visual art training modulated early cortical processing indexed by the N1 and P2 waves. The ANOVA on the N1 mean amplitude (90–130 ms) yielded a significant group × session × sequence type interaction [*F*(2,47) = 4.30, *p* = 0.019, η^2^ = 0.16]. To better understand this three-way interaction, we examined the effects of training as a function of sequence type. For the piano tones, both music and art groups showed larger N1 amplitude at post-test than pre-test (*p* < 0.005 in both cases). For the control group, there was no difference in N1 amplitude between pre-test and post-test. For the vowel stimuli, there was no significant difference in N1 amplitude between pre-test and post-test (*p* > 0.19 in all three groups). Thus, while the N1 amplitude elicited by vowel stimuli was little affected by training, the N1 generated by piano tones was larger at post-test than pre-test in the music and visual art training groups (Figure 3, 4). The interaction between group × session × stimulus type was not significant.

We also observed a main effect of session [*F*(1,47) = 10.15, *p* = 0.003, η^2^ = 0.18], and a session × sequence type interaction [*F*(1,47) = 6.06, *p* = 018, η^2^ = 0.11], with greater increased in N1 amplitude at post-test for the piano tone than for the vowel stimuli. The main effect of stimulus type was significant [*F*(1,47) = 52.19, *p* < 0.001, η^2^ = 0.53], reflecting larger N1 amplitude for deviant than standard stimuli. The interaction between session and stimulus type was also significant [*F*(1,47) = 12.82, *p* = 0.001, η^2^ = 0.21], which was due to greater difference between standard and deviant at post-test than at pre-test.

For the P2 mean amplitude (175–215 ms), the group × session was not significant, nor was the group × session × stimulus type. However, the group × session × sequence type interaction approached the corrected significance level [*F*(2,47) = 3.63, *p* = 0.034, η^2^ = 0.13]. For the piano tones, both music and visual art groups showed larger P2 amplitude at post-test than pre-test (*p* < 0.005 in both cases). For the control group, there was no difference in P2 amplitude between pre-test and post-test. For the vowel stimuli, there was no significant difference in P2 amplitude between sessions (*p* > 0.65 in all three groups). As for the N1 amplitude, the P2 wave elicited by vowel stimuli was little affected by training while the P2 generated by piano tones was larger at post-test than pre-test in the music and visual art training groups (Figure 3, 4).

The omnibus ANOVA on the P2 mean amplitude also revealed a main effect of session [*F*(1,47) = 6.66, *p* = 0.013, η^2^ = 0.12], and an interaction between session and sequence type [*F*(1,47) = 12.80, *p* < 0.001, η^2^ = 0.21]. The main effect of stimulus type was significant [*F*(1,47) = 44.50, *p* < 0.001, η^2^ = 0.49], with the P2 wave being more negative for deviant than for standard stimuli. The sequence × stimulus type interaction was also significant [*F*(1,47) = 21.81, *p* < 0.001, η^2^ = 0.32], reflecting greater deviance-related activity for the vowel stimuli than for the piano tones.

#### Training Effects on MMN

Figure 3, 4 show the mean MMN across groups. For the MMN latency, the group × session interaction was not significant [*F*(2,47) = 1.70, *p* = 0.193, η^2^ = 0.07], nor was the group × sequence type interaction (*F* < 1). The three-way group × session × sequence type interaction was not significant [*F*(2,47) = 1.80, *p* = 0.177, η^2^ = 0.071]. However, the ANOVA yielded a main effect of sequence type [*F*(1,47) = 49.06, *p* < 0.001, η^2^ = 0.51], with the MMN elicited by piano tones (*M* = 150 ms, *SE* = 2.5 ms) peaking earlier than the MMN elicited vowels (*M* = 182 ms, *SE* = 4.1 ms). The main effect of session was also significant [*F*(1,47) = 16.42, *p* < 0.001, η^2^ = 0.23], with earlier peak latency at post- than pre-test.

For the MMN peak amplitude, the group × session interaction was not significant [*F*(2,47) = 2.55, *p* = 0.089, η^2^ = 0.10], nor was the group × sequence type (*F* < 1). However, the MMN was larger for vowel than piano tones [*F*(1,47) = 49.22, *p* < 0.001, η^2^ = 0.51]. All other main effects and interactions were not significant. Thus, neither music nor visual art training had a significant effect on the automatic change detection process as measured by the MMN.

#### Follow-Up Retention

Three months after the end of training, a subgroup of participants (music *N* = 15, visual art *N* = 14) returned for follow-up assessment and evaluation of long-term retention effects. One participant in each group was excluded from the analysis because he/she was missing one of the experimental conditions due to technical problems. The remaining sample comprised 14 from the music group and 13 from the visual art group.

The ANOVA on the N1 mean amplitude with session (pre, post, follow-up) as between-subject factor and sequence (piano tones, vowels) and stimulus type (standard, deviant) as within-subject factors, yielded a significant session × sequence type interaction [*F*(2,50) = 4.47, *p* = 0.016, η^2^ = 0.15]. To better understand this interaction, we performed separate ANOVAs for piano and vowel stimuli. For piano tones, the ANOVA revealed a main effect of session [*F*(2,50) = 11.33, *p* < 0.001, η^2^ = 0.31]. Pairwise comparisons revealed a significant increase in N1 amplitude at post-test and follow-up relative to pre-test (*p* < 0.001 in both cases). There was no difference in N1 amplitude between post-test and follow-up (*p* = 0.144). The group × session interaction was not significant (*F* < 1), nor was the group × session × stimulus type [*F*(2,50) = 1.67, *p* = 0.192, η^2^ = 0.31]. For vowel stimuli, the main effect of session was not significant nor was the group × session interaction (*F* < 1 in both cases). As for the N1, the ANOVA on the P2 mean amplitude yielded a session × sequence interaction [*F*(2,50) = 6.27, *p* = 0.004, η^2^ = 0.20]. For piano tones, the main effect of session was significant [*F*(2,50) = 10.29, *p* < 0.001, η^2^ = 0.29]. Pairwise comparisons revealed larger P2 amplitude at post-test and follow-up than at pre-test (*p* < 0.001 in both cases). There was no difference in P2 amplitude between post-test and follow-up (*p* = 0.51). For vowel stimuli, the main effect of session was not significant (*F* < 1) nor was the group × session interaction (*F* < 1).

In summary, music and visual art training modulated auditory processing, which was retained 3 months after training end.

### Effects of Music and Visual Art Training on ERPs During Visual Go NoGo Task

#### Behavioral Data

All three groups showed ceiling performance on Go trials with few (if any) false positives on NoGo trials (see Table 1). There was no difference between the groups nor between the pre- and post-test sessions. The group × session interaction was not significant for accuracy or response time measures.

**Table 1:** Behavioral results on the GoNoGo task.

*Group*	Session	Group mean response time (ms) and (SE)	Error (%)
*Music*	Pre-training	444 (17)	2.19 (0.62)
	Post-training	427 (15)	2.56 (0.63)
	Follow-up	431 (10)	3.33 (0.62)
*Visual art*	Pre-training	434 (16)	3.33 (0.59)
	Post-training	436 (14)	3.17 (0.59)
	Follow-up	443 (15)	5.50 (2.36)
*Control*	Pre-training	435 (15)	1.71 (0.60)
	Post-training	439 (17)	1.47 (0.61)

#### Training Effects on Visual ERPs

**FIGURE 5:**
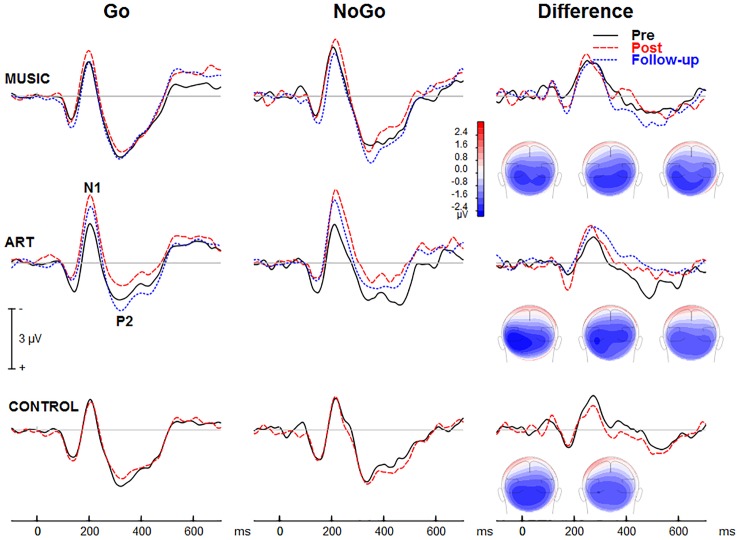
Group mean event-related potentials elicited during the visual GoNoGo task over the right parietal scalp area (i.e., electrode P6). The iso-contour maps (view from the back) show amplitude distribution for the mean voltage between 210 and 280 ms. From left to right, the maps show the distribution at pre-test, post-test, and follow-up, respectively.

Figure 5 shows group mean visual ERPs elicited during Go and NoGo trials as well as the corresponding difference waves at parietal-occipital sites. The scalp-recorded visual ERPs comprised prototypical deflections at ∼100, ∼185, and ∼325 ms after stimulus onset (i.e., P1-N1-P2). The difference wave between Go and NoGo trials reveals processing associated with suppressing the motor response.

We first examined whether music and visual art training modulated early visual cortical processing indexed by the N1 and P2 waves. The ANOVA on the N1 mean amplitude (165–205 ms) yielded a group × session × hemisphere was significant [*F*(2,50) = 4.00, *p* = 0.024, η^2^ = 0.14]. To better understand this three-way interaction, we examined the effects of training as a function of hemisphere. For the left parietal-occipital areas, the music and visual art group showed comparable visual N1 amplitude before and after training (*p* > 0.5 in both cases) whereas the N1 in the control group was larger at post-test than pre-test (*p* < 0.01). For the right parietal-occipital areas, the music and control group showed comparable N1 amplitude at pre-test and post-test, while the N1 amplitude was significantly enhanced in the visual art group (*p* = 0.003). While the N1 amplitude measured over the left hemisphere was little affected by training, the N1 recorded over the right hemisphere was larger at post-test than pre-test in the visual art group only. No other interactions involving group and sessions were significant.

The omnibus ANOVA also showed that the N1 wave was larger at post-test than pre-test [*F*(1,50) = 6.21, *p* = 0.016, η^2^ = 0.11]. The main effect of stimulus type was also significant [*F*(1,50) = 18.78, *p* < 0.001, η^2^ = 0.27], with larger N1 amplitude during the NoGo than Go trials. The latter could reflect an attention-related negativity that overlaps the N1 wave rather than a modulation of the N1 response.

For the visual P2 mean amplitude (305–345 ms), there was no significant interaction between group and session, nor was the group × session × hemisphere interaction significant [*F*(2,50) = 1.35, *p* = 0.269, η^2^ = 0.05]. The main effect of session was not significant [*F*(1,50) = 2.23, *p* = 0.142, η^2^ = 0.04]. However, the session × hemisphere interaction was significant [*F*(1,50) = 4.14, *p* = 0.047, η^2^ = 0.08]. While the P2 measured over the left hemisphere was comparable between pre- and post-training sessions, the P2 recorded over the right parieto-occipital area was larger at post-test.

##### NoGo-related effects

The visual ERPs elicited during the NoGo trials showed a negative displacement, which could be accounted for by attention-related effects superimposed on the N1 and P2. This is best illustrated by the difference wave between Go and NoGo trials. This difference wave revealed a negative component at parietal-occipital sites that peak at about 230–240 ms after stimulus onset. The mean amplitude for 210–280 was used for left (PO7, P5, P7) and right hemisphere (PO8, P6, P8).

**FIGURE 6:**
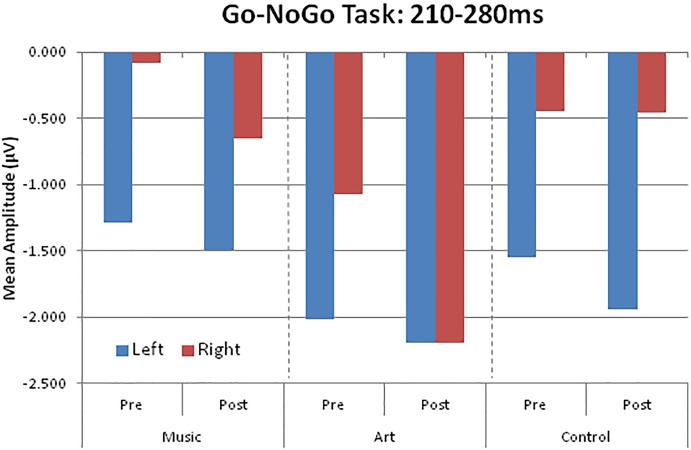
Group mean visual event-related potential amplitudes over left (PO7, P5, P7) and right (PO8, P6, P8) parieto-occipital areas.

The main effect of group was not significant [*F*(2,50) = 1.41, *p* = 0.253, η^2^ = 0.05], nor was the group × session interaction (*F* < 1). However, there was a significant group × session × hemisphere interaction [*F*(2,50) = 6.17, *p* = 0.004, η^2^ = 0.19]. This was due to a shift in the response laterality between the first and second session in the visual art group (Figure 6). That is, at pre-test, the ERP amplitude was greater over the left parietal-occipital sites whereas after visual art training the ERPs showed a more symmetric amplitude distribution over the left and right hemispheres. In the control and music group, the ERP amplitude was larger over the left and right hemisphere during the pre- and post-training sessions.

There was a main effect of session [*F*(1,50) = 6.21, *p* = 0.016, η^2^ = 0.11], with larger amplitude at post-test. The main effect of condition was also significant [*F*(1,50) = 124.47, *p* < 0.001, η^2^ = 0.71], which reflected enhanced negativity generated by the NoGo trials compare to the Go trials.

##### Follow-up retention

We examined whether the changes in laterality persisted 3 months after the end of training. An ANOVA comparing ERPs recorded in all three session in the art group revealed a session × hemisphere interaction [*F*(2,26) = 6.02, *p* = 0.013]. Separate ANOVAs for the left and right hemisphere yielded a main effect of session only for ERPs recorded over the right parietal-occipital area [right hemisphere: *F*(2,26) = 4.35, *p* = 0.029; left hemisphere: *F* < 1]. Pairwise comparisons revealed enhanced negativity at post-test relative both pre-test and follow-up (*p* < 0.05 in both cases). The ERPs at follow-up did not differ from those obtained at pre-test (*p* = 0.693). As during the pre-test, the ERPs recorded at follow-up were larger over the left than the right hemisphere. In summary, art training was associated with increased early attention-related effects over the right parietal-occipital area, which was not retained 3 months after training.

#### ERPs Associated With Response Inhibition

**FIGURE 7:**
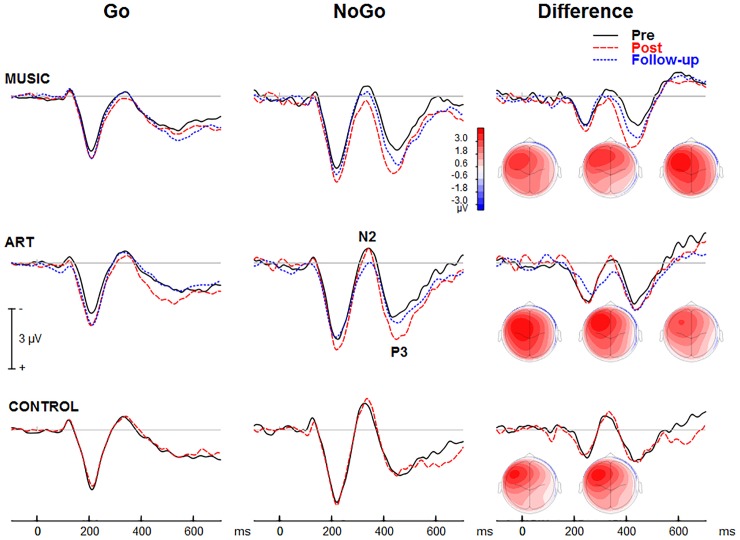
Group mean visual event-related potential elicited during the visual GoNoGo task over the right frontal scalp region (i.e., electrode F2). The iso-contour maps (view from the top) show amplitude distribution for the mean voltage between 375 and 475 ms. From left to right, the maps show the distribution at pre-test, post-test, and follow-up, respectively.

The NoGo trials generated a modulation that peaked at about 425 ms after stimulus onset at fronto-central sites (Figure 7). The group × session × condition × hemisphere interaction was significant [*F*(2,50) = 4.24, *p* = 0.020, η^2^ = 0.15]. To better understand this four-way interaction, we performed separate ANOVAs for each group. In the music group, the main effect of condition was significant [*F*(1,16) = 8.19, *p* = 0.011, η^2^ = 0.339], so was the session × condition interaction [*F*(1,16) = 8.67, *p* = 0.010, η^2^ = 0.351], with greater increased in ERP amplitude at post-test for the NoGo than Go condition. The condition × hemisphere was significant [*F*(1,16) = 13.31, *p* = 0.002, η^2^ = 0.454], reflecting greater difference between Go and NoGo trials over the left hemisphere. The session × hemisphere was not significant (*F* < 1). In the visual art group, the main effect of condition was significant [*F*(1,18) = 21.18, *p* < 0.001, η^2^ = 0.541]. The session × condition was also significant [*F*(1,18) = 8.038, *p* = 0.011, η^2^ = 0.309], with greater increased in ERP amplitude at post-test for the NoGo than Go condition. The session × hemisphere trended toward significance [*F*(1,18) = 3.51, *p* = 0.077, η^2^ = 0.163]. However, the session × condition × hemisphere was significant [*F*(1,18) = 5.78, *p* = 0.027, η^2^ = 0.243]. This was due to greater difference in ERP amplitude between Go and NoGo trials over the left hemisphere after training. In the control group, the main effect of condition was significant [*F*(1,16) = 17.12, *p* = 0.001, η^2^ = 0.517]. However, neither the main effect of session or the session × condition interaction were significant (*F* < 1 in both cases). The condition × hemisphere was significant [*F*(1,16) = 29.04, *p* < 0.001, η^2^ = 0.645], reflecting greater difference between Go and NoGo trials over the left hemisphere. The other main effects or interactions were not significant.

The ANOVA on the mean amplitude (375–475 ms) yielded a main effect of session [*F*(1,50) = 4.37, *p* = 0.042, η^2^ = 0.08], and a main effect of condition [*F*(1,50) = 43.59, *p* < 0.001, η^2^ = 0.466]. That is, the ERP amplitude was more positive at post-test than at pre-test, and more positive for NoGo trials than for Go trials. The session × condition interaction was significant [*F*(1,50) = 12.50, *p* = 0.001, η^2^ = 0.20]. This reflected greater difference between Go and NoGo trials (NoGo ERP effect) at post-test than pre-test. The condition × hemisphere interaction was also significant [*F*(1,50) = 62.18, *p* < 0.001, η^2^ = 0.55]. This was due to larger NoGo responses over the left compared to right frontal scalp region.

##### Follow-up retention

In both music and visual art groups, the NoGo ERP effect was slightly reduced at follow-up compared to post-test, but the difference was not statistically significant. Moreover, the NoGo ERP effect at follow-up did not differ from the effects observed at pre-test. These results suggest that some training effects remained three months after the cessation of training. However, this finding should be interpreted with caution because of the small sample size at follow-up and the lack of difference between pre-test and follow-up session.

## Discussion

Using a partially randomized intervention design, our study reveals changes in older adults’ brain responses following short-term arts training (music and visual art engagement). As such, we provide important new evidence suggesting a causal relationship between these activities and neuroplastic changes in older adults. In the present study, we also observed some training-specific effects using auditory oddball and visual GoNoGo paradigms.

### Arts Training and Auditory Processing

In both groups, we observed increased N1 and P2 amplitude after training, which was retained at least 3 months after the training ended. Although enhanced N1 and P2 amplitude has been observed after training in several studies (e.g., Tremblay et al., 2001; Reinke et al., 2003), this is, to our knowledge, the first study showing training-related increased in N1 and P2 amplitude to untrained stimuli in older adults. In the present study, the effect of training was more pronounced for the piano tones. This difference in training-related N1 effect between piano tones and vowels could be related to familiarity with the material prior to study. Prior research has shown increased N1 and P2 amplitude with increased familiarity (Sheehan et al., 2005; Alain et al., 2015). For most participants, speech sounds such as vowels are over-learned and highly familiar stimuli leaving less opportunity to observe neuroplastic effects. In comparison, the piano tones used in the present study were more novel thereby benefiting more from training. The music group may thus have benefited from exposure to musical sounds; as for the visual art group, it is possible that such training may predispose the brain to be more receptive to new material than over-learned stimuli.

Training-specific changes were not observed in the auditory MMN as response latency peaked earlier at post-test than pre-test in all groups including controls. This suggests that exposure to stimuli at pre-test may be sufficient to facilitate the change detection process. Further research is needed to examine whether more specific training using auditory discrimination tasks would yield greater changes in older adults’ MMN responses.

### Arts Training and Visual Processing

Participants engaged in visual art training showed differences in early attention-related brain processing at posterior parietal-occipital scalp regions. This suggests that visual art training may improve processing of visual features. No such changes in early visual responses were observed in the control group or in participants involved in the music training program. Given that the visuals art group showed changes in both auditory and visual neural processing, these findings reveal an important property about the nature of brain plasticity in aging, showing cross-modality transfer of benefits (here from visual art training to auditory processing).

### Arts Training and Cognitive Control

Following a short 3-month music or visual art training program, functional brain changes in inhibitory control were observed in both training groups. While both forms of training offered similar improvements (i.e., enhanced P3), notably, they also yielded training-specific changes (i.e., differential changes in N1 and P2 for visual art versus music groups). These findings are consistent with earlier reports of training-driven modulations in prefrontal cortical activity of older adults (Voss et al., 2010; Anguera et al., 2013).

At the behavioral level, the music group improved in color naming speed (subtest of the Stroop task) between pre- and post-assessment. This effect of music training could reflect improvement in processing speed. Decline in processing speed has been linked to general cognitive decline (Sliwinski and Buschke, 1999) and may have major consequences for the lives of older adults. Indeed, processing speed plays a critical role in everyday activities by facilitating important cognitive functions like learning and long-term memory, comprehension, decision making, and planning (West, 1996; Baddeley, 2012). Previous correlational studies have reported a link between higher cognitive skills and musicianship (Hanna-Pladdy and MacKay, 2011; Hanna-Pladdy and Gajewski, 2012; Amer et al., 2013). Our study extends these earlier findings by demonstrating a causal relationship between musical training and processing speed in older adults. This finding offers hope for improving the aging process using music intervention strategies that aim to strengthen cognitive skills.

Our results also revealed changes in the neural correlates of inhibitory control during a GoNoGo task following only 3 months of training. In NoGo trials, both training groups, as compared to the control group, showed increased and protracted P3 amplitude over the left hemisphere at post-test while the music group also showed similar effects over the right hemisphere. This is consistent with a prior study in older expert musicians (Moussard et al., 2016). It is noteworthy that the enhancement in NoGo-P3 in left frontal sites actually increased hemispheric symmetry following training. This finding is interesting because such results could be interpreted as a compensatory process in cognitive aging. Neuroimaging work has shown that high-performing older adults recruit additional resources and engage prefrontal cortex bilaterally during demanding tasks (Cabeza, 2002; Cabeza et al., 2002; Grady, 2012; Du et al., 2016). It is possible that training for both groups improved functioning of left frontal brain regions that had declined with age. In Go trials, the music group showed enhanced P3 amplitude at post-training compared to the visual art and control groups. P3 may reflect closure of the inhibition processing of an overt response (Gajewski and Falkenstein, 2013) or the ongoing evaluation of an intention to inhibit (Liotti et al., 2005). Thus, we infer that music training improves supervisory mechanisms that act to ensure desired responses and reinforce interference monitoring (Moreno et al., 2014; Moreno and Farzan, 2015).

Training-specific effects were expressed in P2 and N2 waves, in which we observed seemingly opposite effects for each training group: music students showed increased P2 and decreased N2 amplitudes after training whereas the visual art group showed decreased P2 and increased N2 amplitudes (no-contact controls showed no changes). In our previous study of younger adults undergoing musical instruction (Moreno et al., 2014), larger P2 was also observed in the music group. Taken alongside present findings, this suggests P2 is a common marker of plasticity in the brain’s inhibitory control across the lifespan. The P2 wave is thought to index the ability to construct a representation of the current task context and the associated behavioral response in the early stages of processing (Gajewski and Falkenstein, 2013). Electrophysiological evidence suggests that this early aspect of visual processing has links to higher cognitive functions by facilitating stronger internal representations of behaviorally relevant stimuli (Moreno et al., 2014; Moreno and Farzan, 2015). Here, the larger P2 amplitude in the music group may reflect earlier processing and stronger representation of stimuli and the appropriate response (or non-response) pairing strengthened through training. Our results also showed a reduced N2 after music training, consistent with previous work in young adults (Moreno et al., 2014). N2 has been described as a marker of inhibition and conflict detection/attention load (Nieuwenhuis et al., 2003; Burle et al., 2004; Falkenstein, 2006). We interpret this effect in conjunction with the increased P2 amplitude such that music training facilitates dissociation of desired and undesired stimulus-response planning reflected by increased P2, and this enhanced effect of P2 subsequently reduces the need for cognitive control processes reflected by decreased N2 (Bokura et al., 2001; Nieuwenhuis et al., 2003; Donkers and van Boxtel, 2004; Kok et al., 2004).

On the other hand, the opposite pattern observed in the visual art training group may suggest that this form of training reduced early perceptual demands (reduced P2). This is plausible given that our GoNoGo was a visual paradigm (same domain as visual art training). Subsequent enhancements in later N2 may reflect improved post-perceptual conflict detection and response inhibition. Our findings agree well with existing evidence suggesting an impact of visual art instructions on the ventral visual pathway (Draganski et al., 2004; Op de Beeck and Baker, 2010; Pollmann and von Cramon, 2000) as well as higher-order cognitive brain networks (Chamberlain et al., 2014). Overall, the specific nature of changes induced by music and visual art instructions indicate that these divergent forms of training offer unique influences on brain function as well as enhancements to domain-general skills outside the direct scope of training (i.e., inhibitory control).

### Limitations

A limitation of the current study is that our sample showed a gender imbalanced, which could affect the generalizability of our findings. All three groups comprised a much larger proportion of women than men. In older women, the decrease in estrogen level that occurs post-menopause may affect cognitive functions. Further research should ensure a more balanced representation of men and women and also monitor more closely whether women participants are receiving hormonal replacement therapy.

In the present study, the passive control group was added to assess test–retest effects at post-test (i.e., an improvement that would be due to repeating the same battery of tests a second time). This allowed us to better identify the nature and specificity of improvements at post-test. The purpose of the follow-up assessment was to test whether these training-related improvements (or neural changes) were maintained after training has stopped. Given that the control group did not receive any training, it was not justified to test for maintenance of training-related benefits/changes for this group. Although the control group showed little changes in performance and brain activity, we cannot rule out the possibility that differences would have emerged later due to either repeated testing or normal age-related decline. However, we should note that our study did not aim to directly test whether art training slows down cognitive decline: a much longer follow-up would have been required for this purpose (as healthy older adults do not usually show measurable declines after 3 months). Future longitudinal studies should consider adding additional testing for the control group(s) as well as longer follow-up.

Finally, although quite intensive (three sessions per week), the training program was relatively short (3 months). This may have limited the potential benefits of music training on behavioral performance. Future studies could consider longer periods of training, in order to better reflect the real experience of learning music.

## Conclusion

Collectively, our findings expand current knowledge of neuroplasticity in aging by demonstrating dynamic changes in older adults’ brain function following short-term music and visual arts training. Although the neuroplastic changes remain modest, especially for behavioral measures, our results offer clear causal evidence that the aged brain is more plastic than traditionally thought and suggests new possibilities for cognitive training and rehabilitation. Future studies should use longer training regimens to explore transfer effects to everyday life. With the growth of the aging population in modern society, there is a pressing need to find remedies which counteract cognitive decline, improve the aging process, and ultimately reduce health-related costs. Our study establishes that both visual art and music programs might be effective, engaging, and cost-effective solutions to boost older adults’ brain plasticity. These programs could improve essential skills, bolster inhibitory control, and ultimately improve the quality of life for older adults.

## Ethics Statement

The experimental protocol was approved by the Human Research and Ethics Committee at Baycrest Centre, Toronto, ON, Canada.

## Author Contributions

CA and SM designed the experiments. YL, AM, and GB performed the experiments and collected the data. CA, AM, GB, YL, and JS analyzed the data. CA, AM, GB, YL, and SM interpreted results of experiments. CA, AM, and SM drafted the manuscript. All authors edited and revised the manuscript, and approved the final version of manuscript.

## Conflict of Interest Statement

The authors declare that the research was conducted in the absence of any commercial or financial relationships that could be construed as a potential conflict of interest.
